# A historical and proteomic analysis of botulinum neurotoxin type/G

**DOI:** 10.1186/1471-2180-11-232

**Published:** 2011-10-18

**Authors:** Rebecca R Terilli, Hercules Moura, Adrian R Woolfitt, Jon Rees, David M Schieltz, John R Barr

**Affiliations:** 1 Centers for Disease Control and Prevention, National Center for Environmental Health, Division of Laboratory Sciences, 4770 Buford Hwy, N.E., Atlanta, GA 30341, USA; 2 Association of Public Health Laboratories, 8515 Georgia Avenue, Suite 700, Silver Spring, MD 20910, USA; 3 Oak Ridge Institute for Scientific Education, P.O. Box 117, Oak Ridge, TN 37831, USA

## Abstract

**Background:**

*Clostridium botulinum *is the taxonomic designation for at least six diverse species that produce botulinum neurotoxins (BoNTs). There are seven known serotypes of BoNTs (/A through/G), all of which are potent toxins classified as category A bioterrorism agents. BoNT/G is the least studied of the seven serotypes. In an effort to further characterize the holotoxin and neurotoxin-associated proteins (NAPs), we conducted an *in silico *and proteomic analysis of commercial BoNT/G complex. We describe the relative quantification of the proteins present in the/G complex and confirm our ability to detect the toxin activity *in vitro*. In addition, we review previous literature to provide a complete description of the BoNT/G complex.

**Results:**

An in-depth comparison of protein sequences indicated that BoNT/G shares the most sequence similarity with the/B serotype. A temperature-modified Endopep-MS activity assay was successful in the detection of BoNT/G activity. Gel electrophoresis and in gel digestions, followed by MS/MS analysis of/G complex, revealed the presence of four proteins in the complexes: neurotoxin (BoNT) and three NAPs--nontoxic-nonhemagglutinin (NTNH) and two hemagglutinins (HA70 and HA17). Rapid high-temperature in-solution tryptic digestions, coupled with MS/MS analysis, generated higher than previously reported sequence coverages for all proteins associated with the complex: BoNT 66%, NTNH 57%, HA70 91%, and HA17 99%. Label-free relative quantification determined that the complex contains 30% BoNT, 38% NTNH, 28% HA70, and 4% HA17 by weight comparison and 17% BoNT, 23% NTNH, 42% HA70, and 17% HA17 by molecular comparison.

**Conclusions:**

The *in silico *protein sequence comparisons established that the/G complex is phenetically related to the other six serotypes of *C. botulinum*. Proteomic analyses and Endopep-MS confirmed the presence of BoNT and NAPs, along with the activity of the commercial/G complex. The use of data-independent MS^E ^data analysis, coupled to label-free quantification software, suggested that the weight ratio BoNT:NAPs is 1:3, whereas the molar ratio of BoNT:NTNH:HA70:HA17 is 1:1:2:1, within the BoNT/G progenitor toxin.

## Background

**Figure 1:**
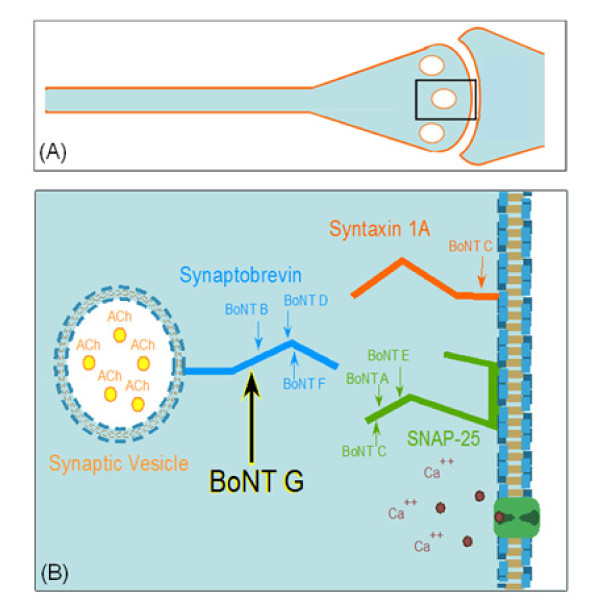
**Graphical representation of the cell and peptide targets of Botulinum neurotoxin**. 1(A) is a representation of the Synaptic cleft where BoNT enters the eukaryotic nerve cell. 1(B) displays the position on the synaptobrevin-2 (VAMP-2) protein where BoNT/G cleaves, stopping the synaptic vesicle from releasing acetylcholine, inhibiting nerve impulse and causing muscle paralysis. In a healthy cell, synaptobrevin-2 on the synaptic vesicle must interact with syntaxin and synaptosomal-associated protein-25 (SNAP-25) on the neuronal membrane for fusion to occur. Fusion allows the nerve impulse to be delivered across the synaptic junction.

*Clostridium botulinum *is the taxonomic designation for at least six diverse species that produce botulinum neurotoxins (BoNTs). This heterologous species is further classified into six metabolically distinct groups (I-VI). The groups include the toxin-forming strains of *C. botulinum, C. butyricum, C. baratii*, and *C. argentinense *[1]. *C. botulinum *is a spore-forming anaerobic bacteria which produces toxins that are lethal to humans and animals, and are classified as category A bioterrorism agents [2,3]. BoNTs target the Soluble NSF Attachment Protein Receptors (SNARE) complex of proteins in the synaptic vesicle and plasma membranes, preventing acetylcholine from being released causing botulism (Figure 1) [3]. Seven immunologically distinct BoNT serotypes (/A through/G) have been described [1,3].

Botulinum neurotoxin G (BoNT/G) is the least studied of the seven serotypes. BoNT/G-producing organisms were first isolated by Gimenez and Ciccarelli in 1969 from soil samples taken from a cornfield in the Mendoza Province of Argentina [4]. The investigators indicated that a novel strain of bacterium produced an antigenically specific, heat-labile botulinum-like toxin that was not neutralized by any of the known botulinum antisera. The antitoxin developed using this strain only neutralized its homologous toxin and showed no activity on any of the other known types of BoNT [4]. Overall, nine strains of type G producing organisms have been isolated, two from Argentina and seven from Switzerland; none of which have ever been clearly implicated as the cause of paralytic illness or death in humans or animals [5].

Type G organisms are historically associated with the *C. botulinum *species, because of their ability to produce botulinum neurotoxin [3,4]. However, it is well known that botulinal toxin production is a poor parameter on which to base species identification and that the *C. botulinum *species is a taxonomic collection of several distinct species [3,5-7]. Type/G producing organisms are classified as *Clostridium argentinense *[5]. This species includes 12 strains of bacteria from the genus *Clostridium*: nine toxigenic strains and three non-toxigenic strains. These strains are genetically and phenotypically distinct from all other strains of *C. botulinum *and other clostridial species [5].

Two of the three non-toxigenic strains were once classified as *C. subterminale*, and the third as *C. hastiforme*. These strains were often reported to cause serological cross-reactions with type/G producing organisms and the BoNT/G protein in ELISA and Fluorescence Resonance Energy Transfer (FRET) detection assays [5,8,9]. The *C. argentinense *species can be distinguished from other asaccharolytic, proteolytic clostridia by a biochemical test that detects the production of a unique derivative of indole [5]. However, to avoid confusion among the medical and scientific communities, *C. argentinense *type/G producing organisms are still referred to as *C. botulinum *type/G [7].

Type/G toxin is produced in culture as a relatively large protein complex (L complex ~500 kDa) consisting of a neurotoxin (BoNT) and three neurotoxin-associated proteins (NAPs): two hemagglutinins (HA17 and HA70) and a nontoxic-nonhemagglutinin (NTNH) component. In addition, there is a gene expression protein (P21) that is responsible for regulating the expression of the four complex proteins. P21, however, is not associated with the toxin complex itself [10,11]. The function of the NAPs has been shown to protect the neurotoxin in harsh environments in order to allow the toxin to enter the synaptic membrane. Once inside the vesicle, the toxin can cleave its specific SNARE complex protein [3,12]. BoNT/G is known to cleave the Synaptobrevin protein (VAMP-2) in the SNARE complex (Figure 1B). It is the only toxin known to cleave at a single Ala^81^-Ala^82 ^peptide bond [13] (Table 1).

**Table 1:** Peptide Cleavage Products for BoNT/B and/G.

	BoNT/B and/G Substrate		Masses
**Intact**	LSELDDRADALQAGASQFESAAKLKRKYWWKNLK		4025
**/B-NT**	LSELDDRADALQAGASQ		1759
**/B-CT**		FESAAKLKRKYWWKNLK	2283
**/G-NT**	LSELDDRADALQAGASQFESA		2281
**/G-CT**		AKLKRKYWWKNLK	1762

The predicted cleavage products and the masses of the substrate and product peptides for both/B and/G are shown. The substrate peptide was derived from the human Synaptobrevin-2 (VAMP-2) protein. Note that/B and/G cleave 4 amino acids apart.

**Figure 2:**
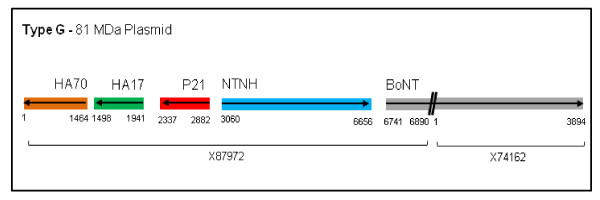
**Schematic of Type G 81 MDa Plasmid**. This is a visual display of the order and direction in which the genes within the BoNT/G complex are associated along the 81 MDa plasmid. NCBI does not have the gene listed under one accession number but rather is split into two: the NAPs X87972 and the toxin X74162.

Type/G-forming organisms have a relatively low toxigenicity, producing only small amounts of toxin in culture. This characteristic makes it difficult to identify type/G organisms in the presence of other species [14]. The toxin requires tryptic activation to be successfully detected *in vitro*; this requirement is also associated with toxins produced by non-proteolytic types/B and/F, as well as all strains of type/E [14]. Regardless of BoNT/G's low toxigenicity *in vitro*, Rhesus monkeys, chickens, and guinea pigs have demonstrated susceptibility to non-activated toxin when BoNT/G has been administered by various routes [15]. In addition, it has been reported that the ability to produce BoNT/G can be lost from toxigenic strains after several culture passages [16]. The loss is thought to occur because the complete nucleotide sequence of the BoNT/G gene, and the NAPs, are found on a 81-MDa plasmid and not on the chromosome [16,17] (Figure 2). Of the seven serotypes, the BoNT/G nucleotide sequence has the most similarity to that of BoNT/B, as previously described [17].

Although BoNT/G is the least studied serotype of *C. botulinum*, previous reports have described a digestion method, two protein detection assays, and an activity detection assay. Hines et al. were the first to apply a proteomics approach for BoNT/G. The authors used a 16-hour digestion method, followed by high-pressure liquid chromatography (HPLC) coupled to mass spectrometry (MS). The method returned limited recovery of peptides and protein sequence coverage. However, it provided enough information to distinguish the proteins associated with the BoNT/G complex [18]. Glasby and Hatheway described the potential use of fluorescent-antibody reagents to identify *C. botulinum *type/G producing strains, but they encountered cross-reactivity issues with similar species of non-toxigenic clostridia [9]. Lewis et al. reported an ELISA BoNT/G protein detection assay that was able to detect low concentrations of the BoNT/G proteins. The assay, however, also suffered from issues of cross-reactivity with similar non-toxigenic *Clostridium *species [8]. Finally, we have previously described a mass spectrometry-based activity detection assay, the Endopep-MS method, which was developed to detect the activity of BoNTs *in vitro *against toxin-specific substrate peptides. This method was successful at detecting all seven BoNT serotypes [19].

Proteomics has been used to study changes after treatment with BoNT/A on suprachiasmatic nucleus [20], on the thyroarytenoid muscle [21], and of C3 exoenzyme from *C. botulinum *[22], but there are very few reports on the BoNT proteome. In the present report, we detail proteomics methods that were successfully applied to the analysis of BoNT/G complex and thus further the understanding of the serotype. We confirmed the detection of toxin activity by use of the Endopep-MS method. The application of a rapid digestion method, coupled with nano ultra-pressure liquid chromatography tandem mass spectrometry (nUPLC-MS/MS), was successful at obtaining a greater percentage of amino acid sequence coverage of each protein associated with the/G complex than was previously reported. In addition, we describe the characterization and relative quantification of the proteins present in the/G complex. We also compare BoNT/G to other BoNT serotypes and discuss the previous literature reports to provide a complete description of the BoNT/G complex.

## Results

### Amino acid sequence comparisons confirmed BoNT/G and/B similarity

**Figure 3:**
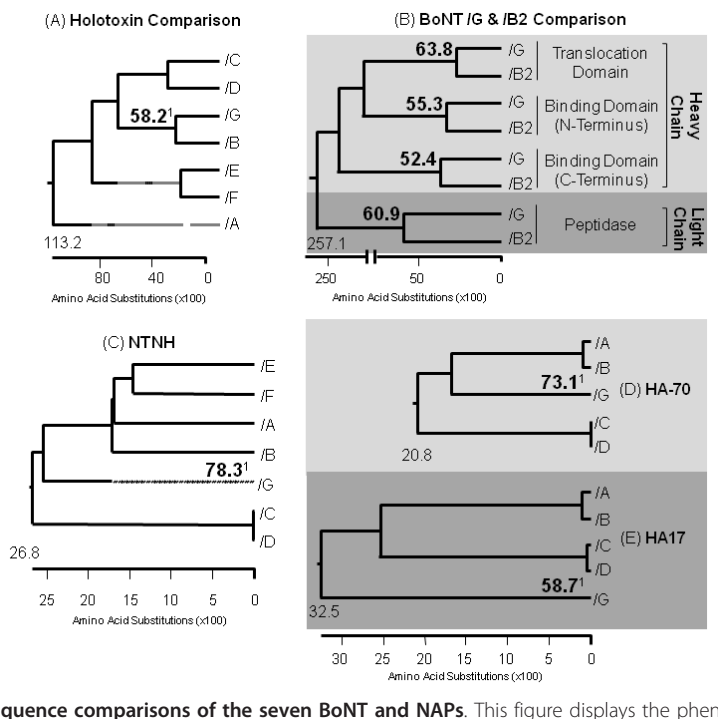
**In-depth protein sequence comparisons of the seven BoNT and NAPs**. This figure displays the phenetic grouping of: (A) the seven serotypes, most common strains, toxin sequences; (B) individually compared toxin domains of/G and the/B2 Prevot strain, the toxin sequence in the/B family that shares the most similarities with/G; (C) the seven serotypes, most common strains, NTNH sequences; (D) the seven serotypes, most common strains, HA70 sequences; and (E) the seven serotypes, most common strains, HA17 sequences. Of the seven serotypes,/G shares the most similarity with the/B serotype. The percent identity shared between each/G and/B protein or domain is highlighted above^1^.

Phenetic analysis of the seven available toxin sequences compared revealed that BoNT/G was the most similar to the BoNT/B Okra and the least similar to BoNT/C Stockholm, with a 58.2% and a 32.9% sequence similarity, respectively (Figure 3A, additional file 1). To determine the extent to which the/G sequence is shared among toxins in the/B family,/G was compared with 22 different/B strains, including subtypes of/B1,/B2,/B3, bivalent (Bv/A and Bv/F), and non-proteolytic/B (np/B). Of the 22 sequences,/G shared the most sequence homology with the/B2 Prevot 25 NCASE strain, with an overall 58.9% sequence similarity (additional file 2). In a focused look at the similarities between/G and the/B2 strain, the individual domains of the toxin proteins were compared. The percent similarity returned for each domain were as follows: peptidase (light chain) 60.9%, translocation (heavy chain) 63.8%, binding N-terminal (NT) (heavy chain) 55.3%, and binding C-terminal (CT) (heavy chain) 52.4% (Figure 3B). Additional comparison of BoNT/G NAPs with the NAPs of the other six serotypes indicated that not only is the type/G toxin sequence the most similar to/B, but the NAPs sequences for both serotypes do as well. The percent similarity returned for the NAPs were as follows: NTNH 78.3%, HA70 73.1% and HA17 58.7% (Figure 3C-D, additional files 3, 4, and 5).

### Gel LC-MS/MS Analysis identified the four main proteins within the BoNT complex

**Figure 4:**
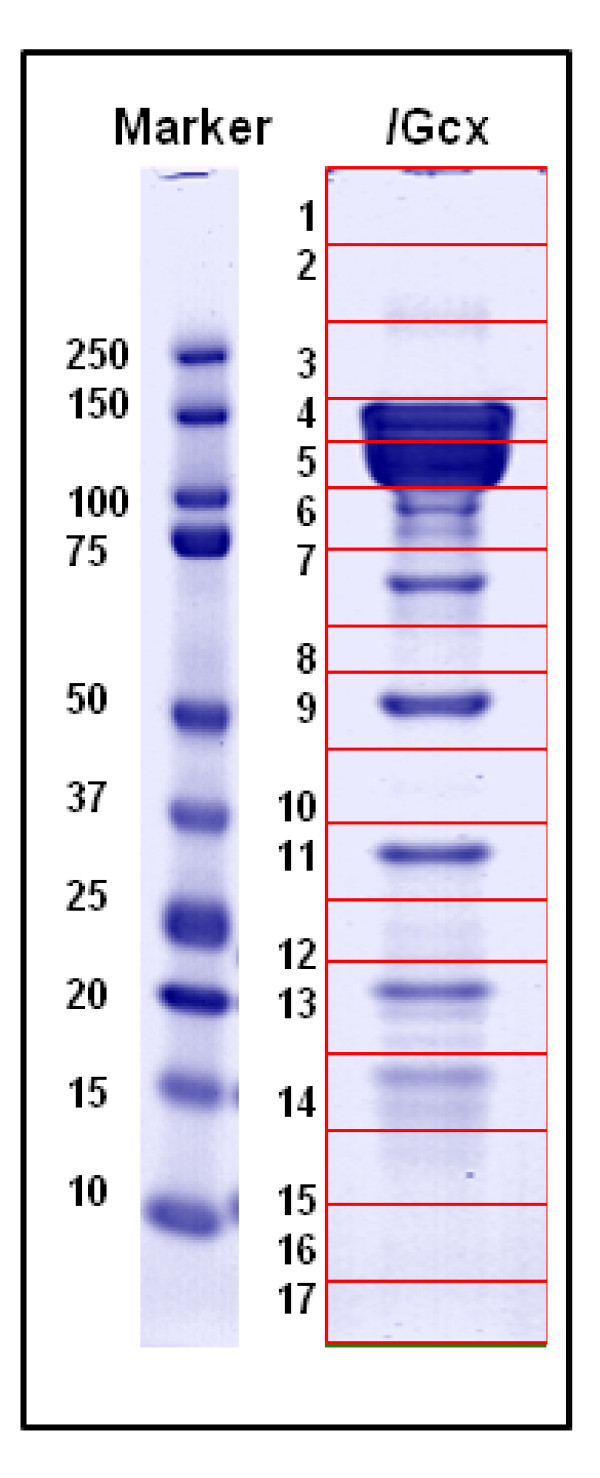
**1D SDS-PAGE and in gel digestion analysis of/G complex**. This image depicts the All Blue standard (Bio-Rad, CA) and the/G complex after staining with GelCode™ Blue Safe Protein Stain (Pierce, IL). The lane of interest was cut into 17 segments, digested overnight, analyzed on a nanoLC-MS/MS system, and identified by use of PLGS protein database searching. The proteins identified were BoNT/G (band 4), NTNH (5); HA70 was identified in three bands (7, 9, and 13) and HA17 in band 14.

Six of the 17 gel slices, tryptically digested overnight and analyzed by use of nLC-MS/MS, returned protein matches with high sequence coverage and a 99% identity confidence when searched by use of PLGS v2.3 and validated with Scaffold v2.1. The four main proteins associated with the botulinum neurotoxin complex were identified in various bands from the gel: BoNT/G, NTNH, HA70, and HA17 (Figure 4).

### In solution Tryptic Digestion Analysis improved protein sequence coverage

**Figure 5:**
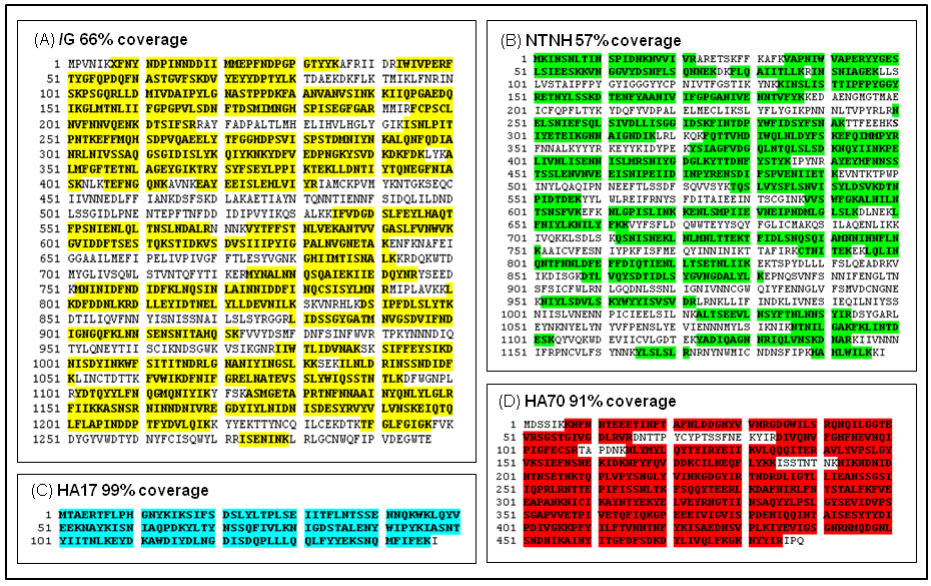
**Sequence coverage returned from in solution tryptic digests**. The four main proteins that are associated with the BoNT/G complex and the percent of each sequence that was returned after digestion are highlighted above. The percent recovery was determined by combining all unique peptides returned from two nanoLC-MS/MS instruments and calculated by use of the number of amino acids recovered vs. total amino acids in the protein sequence. (A) BoNT/G 66% [NCBI, CAA52275] (B) NTNH 57% [NCBI, CAA61228] (C) HA17 99% [NCBI, CAA61226] (D) HA70 91% [NCBI, CAA61225]

The results of the six digests of BoNT/G from both analytical instruments (QTof-Premier and LTQ-Orbitrap) were compiled to determine the greatest percent of sequence coverage of each protein identified: BoNT/G [NCBI, CAA52275], NTNH [NCBI, CAA61228], HA70 [NCBI, CAA61225], and HA17 [NCBI, CAA61226] (Figure 5A-D). The percent recovery was determined by combining all unique peptides identified by both nLC-MS/MS instruments and calculating the ratio of amino acids identified vs. total amino acids in the protein sequence.

### Endopep-MS Analysis confirmed toxin activity

The results of the Endopep-MS experiments conducted through use of various dilutions of BoNT/G indicated that the optimum temperature for/G activity is 42°C, not 37°C as observed with other BoNT serotypes. Additionally, the experiments indicated that the toxin is the most active, or best activated, when first exposed to a short 10 min pulse at 47°C and then continuously incubated at 42°C for 120 hrs. The detection of the 2281 *m/z *(NT) and 1762 *m/z *(CT) product ions in each experiment confirmed that the lots of commercial toxin used were active.

### Relative quantification of type G toxin and NAPs was determined by use of MS^E^

Label-free relative protein quantification was obtained for each component of the type G toxin complex (Table 2). When calculated by weight, the BoNT/G complex contained 30% of toxin, 38% of NTNH, 28% of HA70, and 4% of HA17. These percentages and nanogram amounts indicate that the overall weight ratio of BoNT:NAPs present within the complex is 1:3. The percentages of each molecule present in the complex are as follows: 17.2% of toxin, 23.1% of NTNH, 42.0% HA70, and 17.8% HA17. These percentages and femtomole amounts indicate a 1:1:2:1 BoNT:NTNH:HA70:HA17 ratio, or a 1:4 BoNT:NAPs ratio, of molecules within the complex.

**Table 2:** Relative quantification of Type G toxin and NAPs.

Protein Description	Accession #	Avg Mass (kDa)	Amount OnColumn		% in the Complex	
			**femtomoles**	**nanograms**	**molecules**	**weight**
BoNT/G	CAA52275	149034	110.0	16.4	17.2	30.4
NTNH type G	CAA61228	139083	147.6	20.5	23.1	38.1
HA-70 (III) type G	CAA61225	55791	268.5	14.9	42.0	27.8
HA-17 (II) type G	CAA61226	17372	113.8	1.9	17.8	3.7

The proteins identified in the/G complex, NCBI accession numbers, and average masses are shown, in addition to the calculated amounts on column, femtomoles and nanograms, and the percent of each protein, by weight and molarity, within the BoNT complex.

## Discussion

BoNT/G is the least-studied and the most recently reported of the seven serotypes produced by *C. botulinum*. Although BoNT/G is associated with a distinct species and metabolic group, the toxin shares multiple characteristics with the other six progenitor toxins. The seven serotypes have similar biochemical and molecular mechanisms of cell entry and membrane translocation. They cause disease by inhibiting synaptic transmission as a result of the enzymatic cleavage of the SNARE protein complex. In the present work, we detail the *in silico *comparison of BoNT/G progenitor toxin proteins to the other six serotypes of *C. botulinum*, as well as methods for the digestion, detection, and relative quantification of BoNT/G and its NAPs.

The comparison of the BoNT/G progenitor toxin with the other six serotypes was completed to determine/G's phenotypic relationship with the other BoNTs. In general, past analyses [7,10,23] have included a comparison at the gene level; this study focuses solely on protein level. While comparisons of toxin and NTNH proteins to select serotypes have been previously described [23], a complete comparison of all/G complex proteins (toxin, NTNH, HA70, and HA17) with the other six serotypes has not been previously reported. Phenetic analysis confirmed that the BoNT/G complex of proteins shared the most similarity with the/B serotype (Figure 3C-E), as previously reported [10,23].

To determine the extent of/G's homology to the/B toxin serotype, we completed an in-depth comparison of six/B subtypes, 22 different accession numbers (Figure 3B, additional files 2). The comparison of individual domains--translocation domain, binding domain NT, binding domain CT, and peptidase--revealed the area of the toxin in which/G shares the greatest (translocation domain) and least (binding domain CT) similarity. Overall, each domain compared, between the two toxins, is greater than 50% similar. This comparison helped to determine which substrate peptide would be optimal to test the activity of/G. Although there are no direct indications that sequence similarity would imply overall identical functionality, similar sequences would allow similar crystal structures to form, suggesting similar functionality [24]. It is currently known that both BoNT/B and/G cleave the Synaptobrevin protein;/B cleaves a Gln^76^-Phe^77 ^bond and/G an Ala^81^-Ala^82 ^bond five amino acids downstream (Table 1). Because the cleavage sites of both toxins are relatively near one another--thus the similarity of their binding domain sequences and therefore structures--the same peptide substrate currently used to test/B activity was used to test/G activity [19].

**Figure 6:**
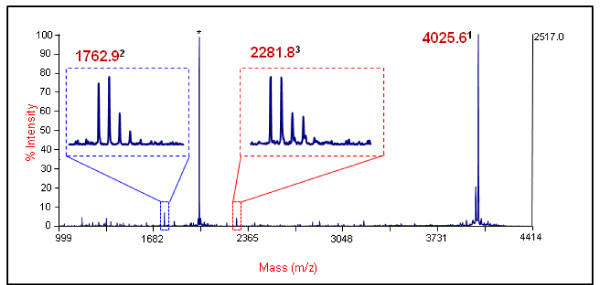
**Endopep-MS method confirmation of commercial BoNT/G activity**. This is a representative spectrum indicating BoNT/G activity on a specific substrate peptide. ^1^Intact substrate, ^2^C-Terminus product mass 1762.9, and ^3^N-Terminus product mass 2281.8. The sequences are listed in Table 1. *Indicates double charged ion of the intact substrate peptide.

In order to confirm that the commercial BoNT/G complex was active and therefore could be considered analogous to the toxin complex found in clinical samples, various dilutions of the commercial toxin were tested using the Endopep-MS method previously described (Figure 6) [19]. In addition to confirming the toxin's activity, the Endopep-MS experiments indicated a new optimum temperature for/G activity. When reactions were pulsed at 47°C for 10 min, followed by incubation at 42°C for at least eight hours--as opposed to 37°C for a minimum of 17 hr--an increase in activity and in the quality of mass spectra produced was observed. Other serotypes of BoNT (/C and/D) are often associated with botulism in animals, avians, equines, and bovines, whose body temperatures are higher than those of humans. BoNT/G has yet to be associated with botulism in a particular organism; however, it is possible that/G would be more effective at causing disease in an organism with a higher body temperature than that of humans, similar to BoNT/C and/D.

Proteomic strategies and analyses used in this study were important to help define the characteristics of proteins associated with the BoNT/G complex. The 1D-SDS PAGE analysis confirmed the presence of the four expected complex proteins (BoNT, NTNH, HA70, and HA17), with relatively high sequence coverage for in gel digestion (Figure 4). As expected the proteins, P21 and HA33, were not identified. P21, a positive regulator of gene expression, lies just upstream of NTNH on the toxin plasmid (Figure 2) [10]. The purpose of P21, in complex development, is not completely understood and previous reports have not identified it as part of the/G complex [11]. HA33, a hemagglutinin component, is not found on the/G plasmid. The lack of evidence of the protein's presence further endorsed the theory that, unlike the other serotypes, HA33 is not associated with the/G complex [10]. Two gel slices (Figure 4; #6 and 11) out of 17 visually had protein but did not return any identifiable peptides when digested and analyzed. This could be due to a number of factors: the protein was relatively difficult to digest, there was not a sufficient amount of protein to digest, the sequence was not present in the database used, or post-translational modifications (PTMs) altered the protein sequence and did not allow for identification. The SDS-Page gel and in gel digestions confirmed visually and analytically which proteins are present in the commercial toxin complex and allowed us to continue to in solution digestions with some prior knowledge of which proteins should be identified.

As anticipated, the same proteins that were identified with the in gel digestions were also identified in the analysis of the in solution digestions. The four main complex components-- BoNT, NTNH, HA70, and HA17--were all identified with high confidence, and returned a large number of peptides. Hines et al. reported the use of a reduction and alkylation overnight digestion method that produced sequence coverages of 16% for BoNT, 10% for NTNH, 38% for HA70, and 49% for HA17 [18]. The method used in our study allowed the recovery of more than four times the sequence coverage for BoNT at 66%, more than five times for NTNH at 57%, and more than double for both HA70 and HA17 at 91% and 99%, respectively.

BoNT complexes are difficult to digest in solution [18]. This rapid high-temperature digestion method does not involve reduction and alkylation, unlike classical methods; instead, it uses an acid labile surfactant to solubilize the hydrophobic proteins. The increased solubility allows a denatured protein to be more susceptible to tryptic digestion, thereby increasing the rate of digestion and the number of tryptic peptides produced [25]. It has also been previously reported that the use of high temperature for a short period of time is the best condition for the enzymatic activity of trypsin [26].

This BoNT complex digestion method, in addition to analysis of the samples on two different electrospray (ESI) MS instruments using data-dependent (DDA) and data-independent MS^E ^analysis, allowed for the detection of a greater number of peptides for each protein, leading to a greater overall sequence coverage than had previously been reported. This sequence coverage lends insight into the complex proteins being studied. A high percentage of sequence coverage indicates that there are few PTMs associated with the proteins, as well as no truncation. The presence of PTMs has been known to compromise protein identification, and truncated proteins do not function as expected.

In addition to providing enhanced sequence coverage, the use of data-independent MS^E ^analysis and label-free quantification software allowed us to relatively quantify the amount of each protein present in the BoNT/G complex (Table 2). This quantification method has the advantage of being able to provide accurate estimates of relative protein abundance (often within 30% of the known values on most identified proteins in a mixture, without the much more rigorous requirements of targeted protein quantification methods. A percentage of abundance (by weight and molecules, separately) of each protein within the complex was determined, as well as an overall weight ratio of BoNT:NAPs and a molecular ratio of BoNT:NTNH:HA70:HA17. Analysis of the individual proteins within the complex illustrated that the weight of the toxin (30.4%) is almost equivalent to that of HA70 (27.8%) and about eight percent less than that of NTNH (38%); whereas HA17 makes up only a minute portion of the overall weight at just 3.7%. Conversely, analysis using molecular amounts indicated that the complex contains an equivalent amount of the toxin, NTNH, and HA17, whereas HA70 is almost twice as abundant. The nanogram and femtomole on column data sets signify a likely overall ratio of 1:3 BoNT:NAPs weight ratio and a 1:1:2:1 BoNT:NTNH:HA70:HA17 molar ratio. As stated earlier, the function of the NAPs has been shown to protect the neurotoxin in harsh environments [12]. Due to this protective ability, in theory, a larger ratio of NAPs:BoNT, ie the greater the number of molecules of NAPs to BoNT, would protect more effectively the toxin from the acidic environment of the stomach. This potentially would increase the toxin's effectiveness at penetrating the mucosa of the intestine and entering the blood stream, increasing the toxin's chances of entering the synaptic cell and causing disease. Knowledge of the stoichiometry of proteins within the BoNT complexes would be useful to further understanding of NAPs significance and toxin potency.

## Conclusions

We have presented a detailed *in silico *comparison of the/G complex of proteins to the other six serotypes in an effort to compare, contrast, and further define the complex's relationship relative to the/B serotype and subtypes within the *botulinum *toxins. Proteomic analyses, consisting of gel electrophoresis, in gel and in solution digestions, and Endopep-MS, confirmed the presence of BoNT, NTNH, HA70, and HA17 proteins and the activity of the commercial/G complex. We were successful at obtaining high sequence coverage for all four complex proteins by using a rapid, high-temperature digestion method and analysing with two different nLC-MS/MS instruments. The efficiency of this method allowed for a greater recovery of protein sequence and further insight into the complex proteins. The use of data-independent MS^E ^data analysis coupled to label-free quantification software suggested that relative quantification of the proteins within BoNT progenitor toxins could be determined and would be very informative to further analysis of *C. botulinum *potency.

## Methods

### Materials and Safety Procedures

We purchased the BoNT/G complex from *C. argentinense *strain 89 from Metabiologics (Madison, WI). The company provided the complex at 1 mg/mL in 50 mM sodium citrate buffer, pH 5.5 and quality control activated. The toxin activity in mouse LD50 or units (U) of specific toxicity obtained from the provider was as follows: [3.3-3.6 × 10^6]. We acquired all chemicals from Sigma-Aldrich (Saint Louis, MO), unless otherwise stated. Los Alamos National Laboratory (Los Alamos, NM) synthesized the substrate peptide used in the Endopep-MS assay. The peptide sequence is listed in Table 1 along with the targeted cleavage products. We followed standard safety handling and decontamination procedures, as described for botulinum neurotoxins [27]. We needed only very low toxin amounts for this work.

### Amino acid sequence comparisons

We carried out all *in silico *work, including the sequence alignments, sequence identities, and phylogenetic trees, using Lasergene software (Protean, EditSeq, and MegAlign^®^--DNA Star Inc; Madison, WI). The alignments followed the Clustal W method [28]. We obtained the toxin protein sequences used for phenetic analysis of the seven BoNT serotypes, the 22 sequences, covering six subtypes, of/B toxin family, and the NAPs (NTNH, HA70 and HA17) of the seven BoNT serotypes from the NCBI protein database (March 2010). For the complete listing of all the accession numbers used in the toxin,/B subtypes, and the NAPs comparison, see additional files 1, 2, 3, 4, and 5.

### One-dimensional sodium dodecyl sulphate/polyacrylamide gel electrophoresis (1D SDS-PAGE)

We added a 4 μL aliquot of [1 μg/μL] commercial BoNT/G complex to 2 μL of NuPAGE^® ^LDS sample buffer and 1 μL NuPAGE^® ^Reducing agent (Invitrogen; Carlsbad, CA) and reduced it by heating at 70°C for 10 min. We cooled and loaded the sample onto a 4-12% NuPAGE^® ^Novex^® ^Bis-Tris mini polyacrylamide gel (Invitrogen) and analyzed it alongside 10 μL of Precision Plus: All Blue and Kaleidoscope protein pre-stained molecular weight markers (Bio-Rad, CA). We performed electrophoresis at 200 V for 35 min, then rinsed the gel 3 × 5 min with dH_2_O and stained it with GelCode™ Blue Safe Protein Stain (Pierce; Rockford, IL) for 1 hr before de-staining overnight in dH_2_O.

### GeLC-MS/MS

#### Sample Excision

We cut the sample lane of interest from a previously run 1D SDS-PAGE gel into 1 × 2 mm slices--17 slices total--and stored the slices at -80°C prior to tryptic digestion.

#### Tryptic Digestion

We lyophilized the individually cut and stored gel slices for 30 min by use of a Centrivap concentrator (Labconco; Kansas City, MO). We added 10 μL of mass spectrometry-grade trypsin (Promega; Madison, WI) to each sample and incubated each sample at room temperature for 5 min. We then added 25 μL of digestion buffer (50 mM ammonium bicarbonate:1 mM CaCl_2_) to each sample and incubated the samples at 37°C overnight.

#### Post-Digestion

We added 5 μL of 0.1% formic acid to the samples for acidification, followed by 2-3 min of sonication to release peptides. We then centrifuged the samples at 12, 100 × g for 10 min to remove insoluble material. We collected the soluble peptide mixtures for nLC-MS/MS analysis.

### nLC-MS/MS analysis

We obtained data by using a nanoAcquity ultra-performance liquid chromatography (nUPLC) coupled to a QTof-Premier MS system (Waters Corp; Milford, MA). We loaded protein digests onto a capillary reverse phase Symmetry C_18 _trapping column and a BEH C_18 _analytical column (100 μm I.D. × 100 mm long, 1.7Å packing; Waters Corp) at a flow rate of 1.2 μL/min. Each sample was separated by use of a 90 min gradient. The mobile phase solvents were (solvent A) 0.1% formic acid (FA; Thermo Scientific; Rockford, IL) in water (Burdick and Jackson; Muskegon, MI) and (solvent B) 0.1% FA in acetonitrile (ACN; Burdick and Jackson). The gradient profile consisted of a ramp from 1%B to 85%B over 82 min, followed by a second ramp to 1%B over 8 min, with data acquired from 5 to 50 min. We analyzed peptides by nano-electrospray on a QTof-Premier hybrid tandem mass spectrometer. The QTof used an MS^E ^(or Protein Expression) method, which involved acquiring data-independent alternating low- and high-collision energy scans over the *m/z *range 50-1990 in 0.6 sec, along with lockmass data on a separate channel to obtain accurate mass measurement.

### In solution Tryptic Digestion for nLC-MS/MS analysis

We completed the tryptic digestions as previously described [25] with few modifications. In all cases, 5 μg of commercial BoNT/G complex was digested, ending with a final digestion volume of 50 μL. All digestions were initially treated with an acid-labile surfactant (ALS) and performed at 52°C for 3 min following the addition of trypsin (Promega; Madison, WI). After acidification, the samples were centrifuged at 12, 100 × g for 10 min to remove insoluble material. The soluble peptide mixtures were then collected for nLC-MS/MS analysis. Once the method was optimized, the experiment was repeated three times for two lots of commercial toxin (six digests total) to confirm that the results were consistent with the proteins that are expected in the toxin complex.

#### nLC-MS/MS analysis

The in solution tryptic digests were analysed by use of two analytical instruments, a QTof-Premier and an LTQ-Orbitrap (Thermo-Finnigan; San Jose, CA), to help to improve the overall protein coverage of the BoNT/G complex. The analyses of digests that used the QTof-Premier were performed initially as described above in the GeLC-MS/MS methods section.

#### LTQ-Orbitrap

Data were obtained by use of an Eksigent 2D nanoLC system (Eksigent Technologies; Dublin, CA) coupled to an LTQ-Orbitrap tandem mass spectrometer. A 365 μm O.D. × 75 μm I.D. fused silica pulled needle capillary (New Objective; Woburn, MA) was packed in house with 10 cm of 5 μm Symmetry 300 reverse phase packing material (Waters Corp). The tryptic digests were loaded directly onto the analytical column without the use of a trap column. The peptide separation was performed over a 120 minute gradient at a flow rate of 400 nl/min. The mobile phase solvents were: (solvent A) 0.2% FA, 0.005% trifluoroacetic acid (TFA) in water, and (solvent B) 0.2% FA, 0.005% TFA in ACN. The gradient was set at 5% B for 5 minutes, followed by a ramp to 30% B over 100 minutes, then a ramp up to 90% B in 5 min and held at 90% B for 2 min before returning to 5% B in 2 min and re-equilibration at 5% B for 20 min. Peptides were analyzed by nano-electrospray on an LTQ Orbitrap hybrid tandem mass spectrometer. The mass spectrometer was programmed to perform data-dependent acquisition by scanning the mass range from *m/z *400 to 1600 at a nominal resolution setting of 60, 000 for parent ion acquisition in the Orbitrap. Then, tandem mass spectra of doubly charged and higher charge state ions were acquired for the top 10 most intense ions. All tandem mass spectra were recorded by use of the linear ion trap. This process cycled continuously throughout the duration of the gradient.

### Endopep-MS analysis of toxin activity

The reactions were performed as described previously [19] with a few modifications. In all cases, the final reaction volume was 20 μL; the final concentration of reaction buffer was 0.02 M Hepes (pH 7.4), 10 mM dithiothreitol, 0.2 mM ZnCl_2_, and 1 mg/mL bovine serum albumin (BSA); and the final concentration of the peptide substrate was 50 picomles/μL. For all experiments, 2 μL [1 μg/μL] of BoNT/G complex was diluted with dH_2_O to various unit (U) concentrations; 1 μL of each dilution was subsequently spiked into 20 μL of reaction buffer and incubated at 37°C, 42°C, or 47°C for 10 min, followed by 42°C for 120 hrs. Time points to gauge the progress of the reaction were taken at 6, 8, 24, 72, and 120 hrs (although in a few cases, a 96 or 144 hr point was taken as a substitute for 120 hrs). 2 μL of each reaction was mixed with 18 μL of α-cyano-4-hydroxycinnamic acid (CHCA) matrix and spotted for analysis by matrix-assisted laser desorption/ionization-time of flight (MALDI-TOF) MS.

#### MS Acquisition

The Endopep-MS reactions were run on a 4800 MALDI-TOF (Applied Biosystems; Framingham, MA). Mass spectra of each sample well were obtained by scanning from 1000 to 4400 *m/z *in MS positive-ion reflector mode. The instrument uses a Nd:YAG laser at 337 nm with a 200 MHz repetition rate, and each spectrum generated was an average of 2400 laser shots. Preceding each run, the instrument was tuned and calibrated for accurate MS analysis by use of a mixture of five peptides: des-Arg1-Bradykinin (*m/z *904.47), angiotensin I (*m/z *1, 296.69), Glu1-fibrinopeptide B (*m/z *1, 570.68), ACTH (1-17)(*m/z *2093.08), ACTH (18-39)(*m/z *2, 465.20).

### nLC-MS/MS and Endopep-MS data processing

#### nLC-MS/MS data

Data obtained from the QTof-Premier were processed by use of Waters' ProteinLynx Global Server (PLGS v2.3; Milford, MA) and searched against a curated *C. botulinum *database consisting of 22, 000 NCBI entries, including the protein standard Alcohol dehydrogenase (ADH, Waters Corp; Milford, MA) and contaminants such as trypsin. Tandem mass spectra were analyzed by use of the following parameters: variable modification of oxidized M, 1% false positive rate, a minimum of three fragment ions per peptide and seven fragment ions per protein, a minimum of 1 peptide match per protein, and with up to two missed cleavages per peptide allowed. Root mean square mass accuracies were typically within 8 ppm for the MS data and within 15 ppm for MS/MS data.

Tandem mass spectra, obtained from the LTQ-Orbitrap, were extracted by Mascot Distiller (Matrix Science; London, UK; v2.2.1.0) and subsequently searched by use of Mascot (Matrix Science; v2.2.0) against a NCBI database consisting of seven million entries. All files generated by Mascot Distiller were searched with the following parameters: 200 ppm parent MS ion window, 0.8 Da MSMS ion window, and up to 2 missed cleavages allowed. Variable modifications for the Mascot searches were deamidation and oxidation.

Scaffold (Proteome Software Inc.; Portland, OR; v2.1.03) was used to validate all MS/MS-based peptide and protein identifications. Peptide identifications were accepted if they could be established at greater than 95.0% probability, as specified by the Peptide Prophet algorithm [29]. Protein identifications were accepted if they could be established at greater than 99.0% probability and if they contained at least two identified peptides. Protein probabilities were assigned by the Protein Prophet algorithm [30]. Proteins that contained similar peptides and that could not be differentiated on the basis of MS/MS analysis alone were grouped to satisfy the principles of parsimony. With the stringent parameters of Peptide Prophet and Protein Prophet, the false discovery rate was zero.

#### Endopep-MS data

The MS Reflector data, obtained from the Endopep-MS reactions, were analyzed by hand. A visual comparison (by an expert researcher) of the intact substrate and its cleavage products was enough to confirm a positive or negative reaction.

### Relative quantification of type G NAPs

The six in solution digestions, three per lot of toxin, of BoNT/G complex were spiked with a known amount of standard yeast ADH digest (100 fMol on column) and analyzed as four technical replicates by use of the QTof-Premier operated in data independent acquisition mode [31,32]. The relative protein quantification of individual replicates was determined based on the average MS signals of the three most intense tryptic peptides per protein, through use of the PLGS Identity^E ^software. Once processed, the data sets were exported from PLGS and clustered according to digestion number for further evaluation by use of Excel (Microsoft Corporation, Redmond, WA). The femtomole and nanograms on column values (Table 2) were calculated by averaging the technical replicates, excluding outliers with 30% or greater variation. These values were then averaged on the basis of lot grouping. The lot grouping averaged values were used to determine a percent by weight, nanograms on column, and a percent of molecules, femtomole on column, of each protein within the BoNT/G complex. In addition, a molar ratio of BoNT:NTNH:HA70:HA17, and BoNT:NAPs, by weight, was determined.

## Authors' contributions

RT helped with the experimental design, carried out experiments, data preparation and *in silico *proteomics analysis, created dendrograms and drafted the manuscript. HM initiated the project, conceived the whole study and experimental design, carried out experiments and contributed to interpretation and writing. AW contributed intellectually to experimental design, data analysis, bioinformatics and manuscript review. JR, DS and JB contributed intellectually to experimental design, data analysis, and manuscript review. All authors read and approved the final manuscript.

## Supplementary Material

Additional file 1 **Protein sequence comparisons of toxin from the 7 BoNT serotypes**. The seven BoNT serotypes toxin sequences (A-G; most common strains) were compared and it was determined that the BoNT/B serotype shared the most sequence similarity to/G. This figure depicts the percent of identity (top to bottom) and percent of divergence (left to right) of the protein sequences compared. Identity equals the percent of similarity the toxin sequences share and divergence the percent of difference between the toxin sequences. Click here for file

Additional file 2 **In-depth comparison of BoNT/G and/B subtypes**. An in-depth comparison of/G and 22/B strains was completed to determine how similar/G was to the/B family. This figure depicts the percent of identity (top to bottom) and percent of divergence (left to right) of the protein sequences compared. Identity equals the percent of similarity the toxin sequences share and divergence the percent of difference between the toxin sequences. Click here for file

Additional file 3 **Protein sequence comparisons of NTNH from all 7 BoNT serotypes**. The seven NTNH serotype toxin sequences (A-G; most common strains) were compared to determine which serotype shared the most sequence similarity to/G. This figure depicts the percent of identity (top to bottom) and percent of divergence (left to right) of the protein sequences compared. Identity equals the percent of similarity the toxin sequences share and divergence the percent of difference between the toxin sequences. Click here for file

Additional file 4 **Protein sequence comparisons of HA70 from all 7 BoNT serotypes**. The seven HA70 serotype toxin sequences (A-G; most common strains) were compared to determine which serotype shared the most sequence similarity to/G. This figure depicts the percent of identity (top to bottom) and percent of divergence (left to right) of the protein sequences compared. Identity equals the percent of similarity the toxin sequences share and divergence the percent of difference between the toxin sequences. Click here for file

Additional file 5 **Protein sequence comparisons of HA17 from all 7 BoNT serotypes**. The seven BoNT serotype HA17 sequences (A-G; most common strains) were compared to determine which serotype shared the most sequence similarity to/G. This figure depicts the percent of identity (top to bottom) and percent of divergence (left to right) of the protein sequences compared. Identity equals the percent of similarity the toxin sequences share and divergence the percent of difference between the toxin sequences. Click here for file
