# Supplementary material for: A historical and proteomic analysis of botulinum neurotoxin type/G
Source: BMC Microbiol. 2011 Oct 18;11:232. doi: 10.1186/1471-2180-11-232 (PMC3215672; doi:10.1186/1471-2180-11-232)
Supplement: Additional file 2 — In-depth comparison of BoNT/G and/B subtypes. An in-depth comparison of/G and 22/B strains was completed to determine how similar/G was to the/B family. This figure depicts the percent of identity (top to bottom) and percent of divergence (left to right) of the protein sequences compared. Identity equals the percent of similarity the toxin sequences share and divergence the percent of difference between the toxin sequences. [file 1471-2180-11-232-S2.PDF]

Additional file 2. In-depth comparison of BoNT /G and /B subtypes

(S-2)

Percent Identity (upper right) / Percent Difference (lower left)

	1	2	3	4	5	6	7	8	9	10	11	12	13	14	15	16	17	18	19	20	21	22	23	
1		58.1	58.3	58.1	58.3	58.2	58.8	58.8	58.8	58.5	58.8	58.8	**58.9**	58.4	58.3	58.3	58.4	58.7	58.4	58.4	58.6	57.6	58.6	**CAA52275 /G strain 89[1]**
2	60.5		99.3	99.3	99.3	99.5	95.3	95.4	95.3	95.4	95.1	95.3	95.3	95.6	93.1	92.7	93.3	96.2	96.1	96.2	96.4	92.6	96.1	ABM73973 /B1 CDC 1656
3	60.1	0.7		99.5	100	99.8	95.7	95.7	95.7	95.7	95.5	95.7	95.7	96.0	93.3	92.9	93.4	96.2	96.0	96.1	96.4	92.8	96.1	AAA23211 /B1 strain
4	60.5	0.7	0.5		99.5	99.8	95.5	95.6	95.5	95.6	95.4	95.5	95.5	96.0	93.3	93.0	93.5	96.1	96.4	96.2	96.3	92.9	96.0	ABM73976 /B1 Hall 6517
5	60.1	0.7	0.0	0.5		99.8	96.7	96.7	96.7	96.7	96.6	95.7	95.7	96.0	93.3	92.9	93.4	96.2	96.0	96.1	96.4	92.8	96.1	BAE48264 /B1 Okra
6	60.3	0.5	0.2	0.2	0.2		95.7	95.8	95.7	95.8	95.6	95.7	95.7	96.0	93.4	93.0	93.6	96.4	96.3	96.4	96.5	93.0	96.3	ABM73983 /B1 CDC 1758
7	59.0	4.9	4.5	4.6	4.5	4.4		99.9	99.8	98.9	99.6	99.8	99.8	98.5	93.9	93.3	94.2	95.4	95.1	95.2	95.4	93.0	95.4	ABM73972 /B2 ATCC 7949[2]
8	59.0	4.8	4.4	4.6	4.4	4.3	0.1		99.9	99.0	99.7	99.9	99.9	98.5	94.0	93.3	94.3	95.4	95.1	95.2	95.4	93.0	95.4	ABM73975 /B2 Smith L-590[2]
9	59.1	4.9	4.5	4.6	4.5	4.4	0.2	0.1		98.9	99.6	100	99.8	98.5	94.0	93.3	94.3	95.4	95.0	95.1	95.4	93.0	95.3	BAC22064 /B2 strain[2]
10	59.6	4.8	4.5	4.6	4.5	4.3	1.1	1.0	1.1		98.7	98.9	98.9	98.3	94.0	93.3	94.3	95.4	95.1	95.2	95.4	93.0	95.4	ABM73978 /B2 6291
11	59.1	5.1	4.6	4.8	4.6	4.6	0.4	0.3	0.4	1.3		99.6	99.6	98.2	93.8	93.2	94.1	95.3	95.0	95.0	95.3	92.9	95.2	ABM73988 /B2 CDC1828[2]
12	59.1	4.9	4.5	4.6	4.5	4.4	0.2	0.1	0.0	1.1	0.4		99.8	98.5	94.0	93.3	94.3	95.4	95.0	95.1	95.4	93.0	95.3	ABM73984 /B2 Prevot 59[2]
13	58.8	4.9	4.5	4.6	4.5	4.4	0.2	0.1	0.2	1.1	0.4	0.2		98.5	93.9	93.3	94.2	95.4	95.0	95.1	95.4	93.0	95.3	**ABM73985 /B2 Prevot 25 NCASE[1]**
14	59.8	4.6	4.1	4.1	4.1	4.1	1.6	1.5	1.5	1.7	1.8	1.6	1.6		93.7	93.2	94.0	95.7	95.6	95.5	95.7	93.0	95.7	ABM73977 /B3 CDC 795
15	60.1	7.2	7.1	7.0	7.1	6.9	6.4	6.3	6.3	6.3	6.5	6.3	6.4	6.6		98.8	99.1	93.0	98.9	92.8	93.0	98.8	92.9	ABM73987 /npB Eklund 17B
16	60.1	7.7	7.5	7.4	7.5	7.3	7.1	7.0	7.0	7.0	7.2	7.0	7.1	7.2	1.3		98.4	92.4	92.5	92.4	92.4	97.5	92.3	ABM73979 /npB 10068
17	59.8	7.1	6.9	6.8	6.9	6.7	6.1	6.0	6.0	6.0	6.1	6.0	6.1	6.2	0.9	1.6		93.0	93.0	92.9	93.0	97.9	93.0	ABM73971 /npB ATCC 17844
18	59.3	3.9	3.9	4.0	3.9	3.7	4.7	4.7	4.8	4.7	4.9	4.8	4.8	4.4	7.4	8.0	7.3		99.5	99.7	99.8	92.4	99.9	ABM73986 /BivBa Ba207[3]
19	59.8	4.0	4.1	3.7	4.1	3.8	5.1	5.1	5.1	5.1	5.2	5.1	5.1	4.6	7.5	7.9	7.4	0.5		99.8	99.3	92.3	99.4	AAK97132 /BivB strain 1436
20	59.8	3.9	4.0	3.1	4.0	3.7	5.0	5.0	5.1	5.0	5.1	5.1	5.1	4.6	7.6	8.0	7.5	0.3	0.2		99.5	92.3	99.6	ABM73974 /BivBf Bf258
21	59.5	3.7	3.7	3.8	3.7	3.6	4.7	4.7	4.8	4.7	4.9	4.8	4.8	4.4	7.4	8.0	7.3	0.2	0.7	0.5		92.4	99.8	AAL11499 /BivB Cryptic neurotoxin
22	61.6	7.8	7.6	7.5	7.6	7.4	7.4	7.3	7.3	7.6	7.5	7.3	7.4	7.4	1.3	2.5	2.1	8.0	8.1	8.2	8.0		92.3	CAA50482 /npB Eklund 17B-ATCC25765[4]
23	59.5	4.0	4.0	4.1	4.0	3.8	4.8	4.8	4.9	4.8	5.0	4.9	4.9	4.5	7.5	8.1	7.4	0.1	0.6	0.4	0.2	8.1		CAA73968 /BivBf CDC3281- ATCC4375

[1] Indicates the sequences that are the most similar

[2] 2nd most similar to /G

[3] 3rd most similar to /G

[4] Sequences that are the least similar to /G
