# Supplementary material for: A historical and proteomic analysis of botulinum neurotoxin type/G
Source: BMC Microbiol. 2011 Oct 18;11:232. doi: 10.1186/1471-2180-11-232 (PMC3215672; doi:10.1186/1471-2180-11-232)
Supplement: Additional file 3 — Protein sequence comparisons of NTNH from all 7 BoNT serotypes. The seven NTNH serotype toxin sequences (A-G; most common strains) were compared to determine which serotype shared the most sequence similarity to/G. This figure depicts the percent of identity (top to bottom) and percent of divergence (left to right) of the protein sequences compared. Identity equals the percent of similarity the toxin sequences share and divergence the percent of difference between the toxin sequences. [file 1471-2180-11-232-S3.PDF]

Additional File 3. Protein sequence comparisons of NTNH from all 7 BoNT serotypes.

Percent Difference	Percent Identity: 1	2	3	4	5	6	7	
1		72.3	78.3	66.9	66.9	62.4	65.4	CAA61228 /G strain 89
2	34.5		83.1	65.6	65.6	68.5	77.4	ABD65471 /A Hall strain
3	25.7	19.3		68.3	68.6	62.2	65.3	BAE48263 /B Okra strain[1]
4	43.5	45.8	41.1		99.7	58.7	59.7	BAE47783 /C Stockholm strain
5	43.5	45.8	41.1	0.3		58.2	59.7	BAA75083 /D strain 1873
6	51.7	40.8	52.1	60.4	60.4		75.8	ACD52603 /E Alaska strain[2]
7	46.2	26.9	46.5	57.2	57.2	29.2		CAA67511 /F Langeland strain

[1]Indicates the sequences that are the most similar
[2]Sequences that are the least similar to /G
